# Detection and genomic characterization of a travel-associated ECSA lineage chikungunya virus infection in Mexico

**DOI:** 10.1128/asmcr.00035-26

**Published:** 2026-07-21

**Authors:** Daniel Canul Canul, K. Jacqueline Ciau-Carrillo, J. Reyes Canche Pech, Cesar Cañas-Alamilla, Jesús Kú-Cachón, Ana Osorio-Medrano, Maria E. Lopez-Novelo, Igrid García González, James Earnest, Daniel Limonta, Norma Pavia-Ruz, Pablo Manrique-Saide, Fabián Correa-Morales, Guadalupe Ayora Talavera, Laura Conde-Ferraez, Azael Che-Mendoza, Jorge Palacio-Vargas, Rafael Valdez-Vázquez, Julissa Guzman-Hernandez, Carlos Frederico Campelo de Albuquerque e Melo, Gonzalo Vazquez-Prokopec, The Arbovirus Co-Circulation (ARCC) Research Consortium, Tierra Smiley, Tetyana I. Vasylyeva, Marina Escalera-Zamudio, Miguel A. Garcia-Knight, Henry Puerta-Guardo

**Affiliations:** 1 Universidad Autónoma de Yucatán, Centro de Investigaciones Regionales Dr. Hideyo Noguchi, Merida, Yucatan, Mexico; 2 Biomédicos de Mérida, Laboratorios Clínicos, Mérida, Yucatan, México; 3 Burnett School of Biomedical Sciences, University of Central Florida, Orlando, Florida, USA; 4 Universidad Autónoma de Yucatán, Unidad Colaborativa de Bioensayos Entomológicos (UCBE) 27778, Merida, Yucatan, Mexico; 5 Secretaría de Salud México, Centro Nacional de Prevención y Control de Enfermedades (CENAPRECE), Mexico City, Mexico; 6 Pan American Health Organization 50514, Washington, DC, USA; 7 Department of Environmental Sciences, Emory University 1371, Atlanta, Georgia, USA; 8 Joe C. Wen School of Population and Public Health, University of California 8788, Irvine, California, USA; 9 Genetics Institute, University College London 4919, London, United Kingdom; 10 Instituto de Investigaciones Biomédicas, Universidad Nacional Autónoma de México 7180, Mexico City, Mexico; 11 Wellcome Sanger Institute 47665, Hinxton, England, United Kingdom; Rush University Medical Center, Chicago, Illinois, USA

**Keywords:** chikungunya virus, ECSA, phylogenetics, arbovirus, Mexico

## Abstract

**Background:**

In 2013, chikungunya virus (CHIKV), a re-emerging *Aedes*-borne virus, was introduced into the Americas. This led to synchronous epidemics across the region associated mainly with the Asian lineage, which eventually subsided. Resurgent outbreaks have been recorded since, principally in South America, largely driven by the East-Central-South-African (ECSA) lineage. In 2025, more than 300,000 CHIKV suspected cases were reported in Brazil and Cuba.

**Case Summary:**

In November 2025, a healthy adult male traveling from Cuba arrived in Merida, Mexico, and shortly after presented febrile symptoms consistent with an arboviral infection. CHIKV infection was diagnosed by RT-qPCR. Though the infection was mild, the patient developed a rash on the abdomen and neck that persisted for up to a month, with further inflammation of the joints of the left leg. Phylogenetic analysis of the viral genome indicated placement within the ECSA lineage, clustering with other contemporaneous virus genomes sampled from Brazil that belong to a recently described clade II within the country, in which viral genomes from Cuba also cluster.

**Conclusion:**

We identify a travel-associated ECSA lineage CHIKV case in Mexico. This viral lineage has not previously been detected in the country. This finding highlights the risk for subsequent local transmission and is consistent with reports of the presence of this lineage in Cuba. Ten years since the last CHIKV epidemic in Mexico, strengthened surveillance is required to anticipate potential local outbreaks within the region.

## INTRODUCTION

Chikungunya (CHIKV; genus *Alphavirus*, family *Togaviridae*) is a re-emerging *Aedes*-borne virus that causes an acute febrile illness (CHIKF) with an incubation period of typically 3–7 days. The disease is generally self-limiting but can result in severe polyarthralgia, with around 44% of all cases developing chronic symptoms; in rare instances, infection may result in neurologic complications and death (1). Around 35 million CHIKV infections occur each year globally, including epidemics across tropical and subtropical regions primarily driven by the *Aedes aegypti* mosquito vector. The Americas have been particularly affected, with up to 2.5 million reported cases yearly (2). In areas endemic for dengue and Zika viruses, which are transmitted by the same mosquito vector, overlapping case definitions and shared clinical symptoms highlight the importance of accurate diagnosis supported by genomic surveillance (3).

Three major CHIKV lineages have been described: the West African (WA), Asian, and East-Central-South-African (ECSA). The WA lineage has been historically associated with enzootic transmission and local outbreaks in Western Africa, while the ECSA and Asian lineages have widely expanded into new geographic regions since their emergence (3). In 2013, the Asian lineage was introduced into the Americas, leading to explosive epidemics that affected over 50 countries (4). In parallel, the ECSA lineage was introduced into Brazil in 2014 and became endemic, with a subsequent diversification into two distinct co-circulating subclades (5). In 2025, more than 110,000 (confirmed and suspected) CHIKV cases were reported across the Americas, with over 38,000 suspected cases from Cuba (6). However, genomic surveillance remains highly heterogeneous across regions, with most genomes from this recent outbreak being sampled from Brazil, whilst a limited number are currently available from Cuba and the region (7).

Between December 2025 and May 2026, Mexico reported 31 confirmed CHIKF cases in the southern states of Quintana Roo (18 cases), Yucatan (1 case), and Chiapas (12 cases) (8). These states are considered risk areas for multiple *Aedes*-borne arboviruses, with the state of Yucatan recognized as a transmission hotspot (9, 10). Moreover, recent serological surveys indicate potential exposure to CHIKV among children aged 2–15 years residing in urban areas of Yucatan, with an IgG seroprevalence of up to 24%, suggestive of ongoing or recent local exposure (11).

Despite an extensive and recent spread of CHIKV across the Americas, its geographic distribution remains poorly understood. Moreover, the extent to which virus diversity reflects cryptic circulation versus repeated introduction events is unclear. Here, we characterize a travel-associated CHIKV case from Mexico. Through phylogenetic placement of the complete viral genome within a broader context of current global CHIKV genetic diversity, we provide new insights into the potential expansion of the ECSA lineage into the Caribbean and potentially Mexico, highlighting surveillance priorities with implications for outbreak preparedness.

## CASE PRESENTATION

**Fig 1:**
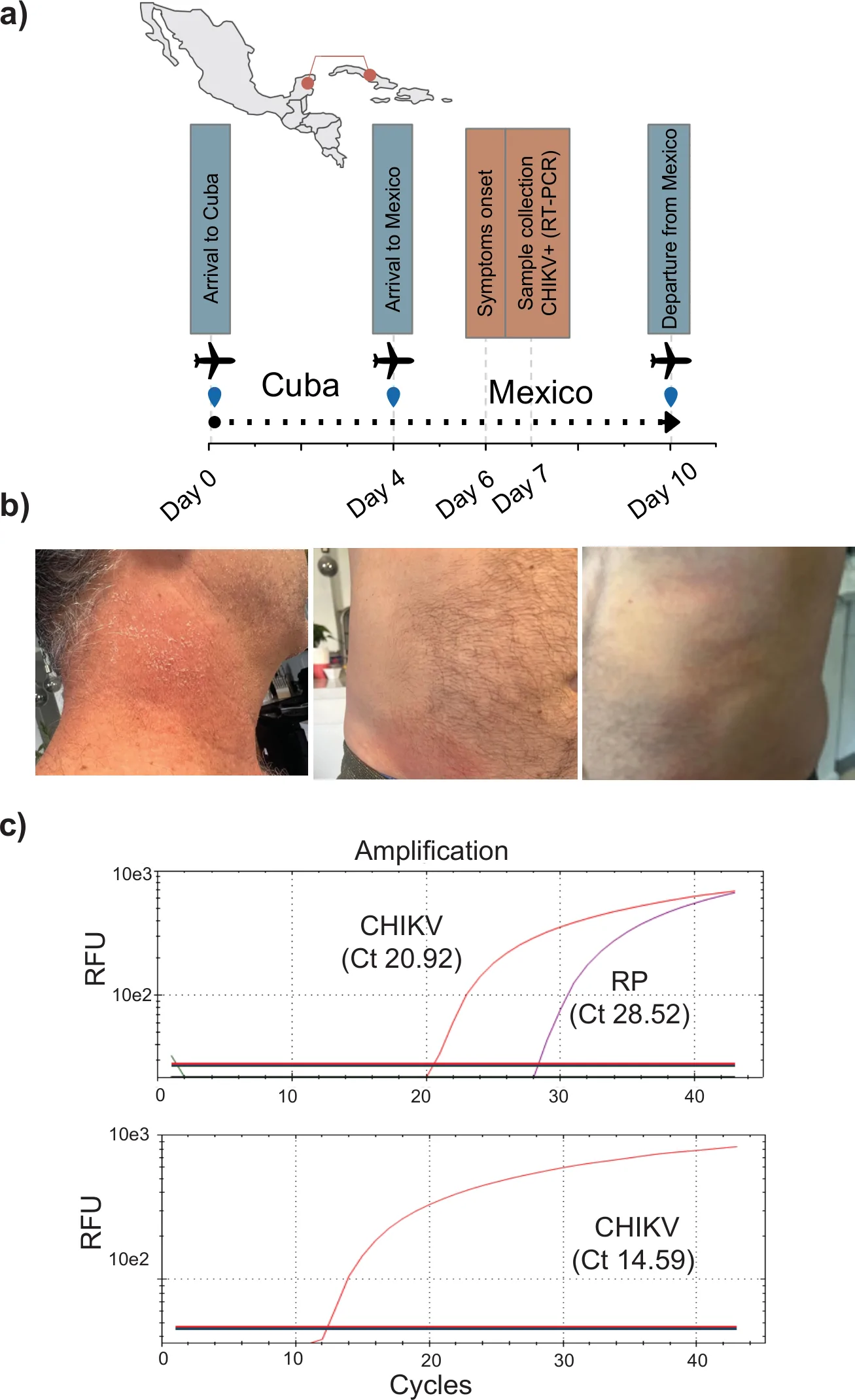
Travel-associated CHIKV case confirmation by detection of CHIKV RNA from plasma and a cell culture isolate. (**a**) Timeline for travel-associated CHIKV case diagnosis and symptom development. Geographic map made with Natural Earth. (**b**) Maculopapular rash on the posterior neck and left and right sides of the abdomen rash. (**c**) RT-qPCR amplification plots for the detection of CHIKV RNA from plasma (top panel) and from cell supernatant derived from Vero-infected cells (lower panel). The RNase P (RP) human gene was used as a reference control for amplification. RFU, relative fluorescent units.

A healthy adult male in his 50s traveled from Miami, Florida, USA, to Havana City, Cuba, in late October 2025 (noted here as day 0), after which he flew to Merida, Mexico (day 4). During his stay in Cuba, no travel outside the urban area occurred. Two days following arrival in Mexico (day 6), he developed a mild fever and cold-like symptoms, including headache, myalgia, arthralgia, and malaise (Fig. 1a). On day 7, the patient reported increased fever (maximum 39°C), pruritus, and an asymmetric maculopapular rash on the posterior neck and abdomen (Fig. 1b). CHIKV infection was diagnosed by RT-qPCR (Ct 20.92), as previously described (11) (Fig. 1c). The patient reported rash persistence on the neck for over 2 weeks, while the rash on the abdomen persisted for 1 month; inflammation and swelling on the ankle and popliteal fossa on the left leg were documented. No further symptoms were reported.

**Fig 2:**
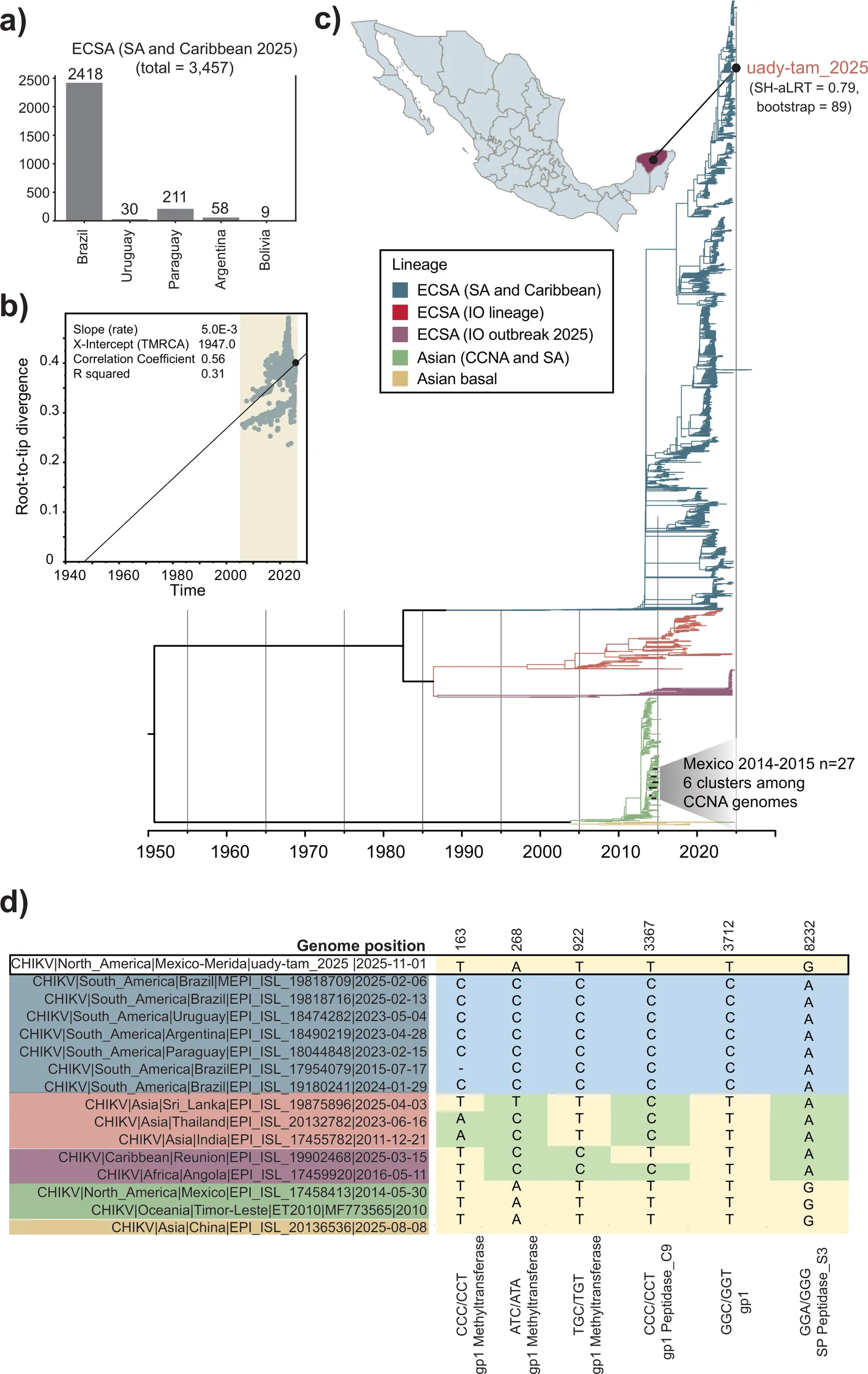
Phylogeny of CHIKV and analysis of signature synonymous mutations. (**a**) The number of genomes included from distinct countries in South America (SA) are indicated. For clarity, the genome sequenced in this study from a travel-associated case linked to Cuba is not shown. (**b**) Root-to-tip regression used to assess the temporal signal of the data set. The shaded region highlights the tip sampling period. (**c**) Maximum likelihood time-scaled phylogenetic analyses of 4,651 CHIKV genomes, with branches colored according to major CHIKV lineages and sublineages. The maximum-likelihood (ML) tree was reconstructed using IQ-TREE 2 v2.2 under a GTR + G substitution model, and a time-scaled phylogeny was inferred using TreeTime v0.9.5 informed by tip dates. The uady-tam_2025 genome is indicated with a black dot on the tree within the ECSA lineage, in a subclade that includes genomes from SA and the Caribbean. Genomes sampled from Mexico during the 2014–2015 CHIKV epidemic clustering with the Asian lineage, with genomes from the Caribbean, Central America, and North America (CCNA), are also shown. Geographic map made with Natural Earth. (**d**) Single nucleotide polymorphisms (SNPs) representing synonymous mutations in uady-tam_2025 identified by pairwise comparison relative to the genome positions in the CHIKV reference genome (NC_004162). Lineage-specific SNP patterns highlighting the differences between uady-tam_2025 and contemporary ECSA genomes are shown in blue. SNP comparison with other more distantly related lineages is further highlighted in light yellow or green. Non-synonymous mutations absent from the parental clade are not shown in the figure and are detailed in the text. IO, Indian Ocean; TMRCA, time to most recent common ancestor.

**Fig 3:**
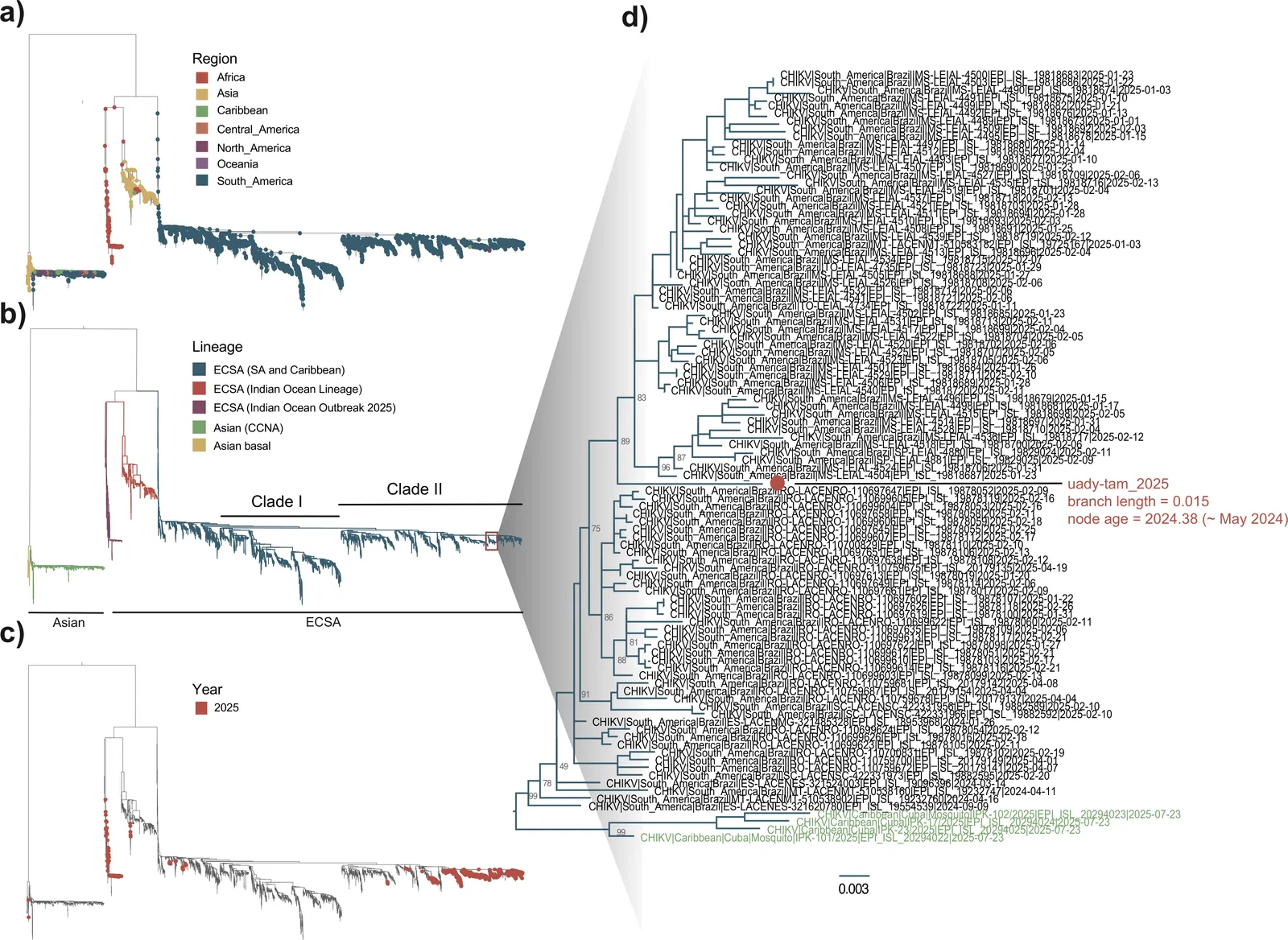
Annotated maximum-likelihood tree and high-resolution placement of uady-tam_2025 in the context of CHIKV genomes sampled in Cuba and globally. Maximum likelihood phylogenetic analyses were carried out on an updated data set, using the same parameters as the primary data set, in which four recently available partial CHIKV genomes from Cuba were added. The ML tree is annotated by (**a**) region, (**b**) lineage (indicating recently described clades of CHIKV reported in Brazil [5]), and (**c**) year of sampling (2025). For clarity, the location of uady-tam_2025 on the tree and clades corresponding to CHIKV ECSA clades I and II from Brazil are only indicated in panel b. (**d**) A detailed zoom into an ECSA clade that includes genomes from Brazil and Cuba (green taxa) is shown. A bootstrap support value (1000 bootstrap replicates) for key nodes is indicated, and the maximum-likelihood point estimate for the node age is indicated for the node/branch, corresponding to uady-tam_2025. The scale bar represents nucleotide substitutions per site.

To further characterize the infection, full viral genome sequencing and phylogenetic analyses were carried out. Briefly, RNA was extracted from plasma and converted to cDNA. Multiplex PCR was used to generate tiling amplicons (12). Sequencing libraries (Native Barcoding Kit, NBD104, Oxford Nanopore Technologies, Oxford, UK) were built and sequenced on a MinION sequencer. Base-called raw reads were assembled to generate a consensus genome sequence (named hereafter uady-tam_2025) using Genome Detective (13), showing 94.9% genome coverage. Virus isolation was further carried out in cultured Vero cells (Fig. 1c) with subsequent sequencing. Both consensus genomes showed 99.8% nucleotide identity. The viral genome from plasma was aligned using MAFFT v7 (14) together with all publicly available, non-embargoed, high coverage (<5% Ns) CHIKV genomes (except WA lineage genomes) with complete coding sequences derived from human cases with complete collection dates (*n* = 4,651), including 3,456 genomes assigned to the ECSA lineage sampled across the Americas (Fig. 2a). Four recently available partial CHIKV genomes from mosquitos and humans from Cuba were also included in an updated data set and analysis (Fig. 3).

A root-to-tip regression plot indicated a temporal signal in the data set (R^2^ = 0.31, correlation coefficient = 0.56, Fig. 2b). A time-scaled maximum likelihood (ML) phylogenetic tree (TreeTime v0.9.5), inferred from a tree reconstructed using IQ-TREE 2 v2.2 under a GTR + G substitution model, revealed that uady-tam_2025 falls within the ECSA lineage (Fig. 2c). The genome clustered with moderate support (SH-aLRT = 0.79, bootstrap = 89) with genomes collected between January and February 2025 from Brazil that belong to a recently described clade II within the country (5) (Fig. 3a through d). Comparison of the branch length (0.015 s/s) leading to uady-tam_2025 to the most closely related sequences suggests continued virus evolution and substantial under-sampling of related genomes (Fig. 3d), while the ML estimate of the node age (2024.38, ~May 2024) suggests possible introduction and divergence of the lineage in Cuba after this date. Of note, the phylogenetic placement of a cluster of virus genomes from Cuba sampled in July 2025 within the clade II of Brazilian CHIKV genomes (Fig. 3d) confirms the local transmission and circulation of the ECSA lineage in the country.

Although uady-tam_2025 and the available Cuban genomes do not appear to share direct ancestry, they form part of the same broader phylogenetic cluster, supporting a co-circulation of related viral diversity across these regions. By contrast, all CHIKV genomes collected during the 2014-2015 epidemic in Mexico fall within the Asian lineage (Caribbean, Central America, and North America Clade; CCNA) (Fig. 2b), in line with previous reports (10, 15). Within a broader phylogenetic context, our analysis also points out to a synchronous circulation of multiple CHIKV lineages as of 2025, each associated with geographically distinct outbreaks. Particularly, multiple ECSA sublineages have given rise to large ongoing and/or recent outbreaks in Indian Ocean islands, southern Asia, China, and South America (Fig. 3b) (16–18).

We further determined the mutational profile of uady-tam_2025 and found that it contains all clade-defining mutations recently described for clade II from Brazil (nsP2-P352A [C2735G], nsP4-A43V [C5793T], and E1-T288I [C10856T]) (5). It also carries mutation E1-T98A (G10285A), associated with enhanced replication in *Aedes albopictus* within the background of mutation E1-A226V, although the latter has not been detected in any ECSA genomes sampled from the Americas (19). In line with the branch length observed in Fig. 3d, uady-tam_2025 also presents multiple synonymous mutations absent from the parental clade (Brazil ECSA clade II) but found in other more distantly related lineages, particularly the Asian lineage (Fig. 2b), as well as several non-synonymous substitutions (nsP1-R144K [G507A], nsP2-L494I [C3161A], nsP3-L434P [T5376C], nsP3-E333D [A5074T], and nsP3-D518Y [G5627T]). These mutations may be indicative of convergent evolution, reversions, or retention of ancestral characters and may serve as sublineage-defining mutations as more genomic data from the region becomes available.

## DISCUSSION

We characterized a CHIKF case in Mexico with a travel history from Cuba, a country with an active large-scale ongoing CHIKV outbreak. Together with recent reports of confirmed cases in Mexico, these raise important public health concerns. CHIKV can be introduced into new areas through viremic travelers, where local transmission may be established in the presence of competent *Aedes* mosquitoes and a susceptible population (9–11, 20–22). The introduction of the ECSA lineage into Mexico, with evidence for heightened virulence compared with the previously circulating Asian CCNA clade (15), has not previously been documented. Previous studies have shown that CHIKV spread dynamics are linked to human mobility along migration routes from Central America into Mexico (10). In this context, this newly characterized case highlights the importance of international air travel through major tourist hubs as a pathway for new viral introduction events.

With 10 years having elapsed since Mexico’s major chikungunya epidemic (2014–2015), population-level immunity is likely to have waned (20), increasing susceptibility to renewed transmission in *Aedes*-endemic urban settings. Moreover, it remains unclear whether immunity acquired against the previously circulating Asian CCNA clade confers effective protection against the ECSA lineage (23). Thus, both a declining herd immunity and potential antigenic differences between lineages could increase susceptibility and facilitate a large-scale epidemic.

The current sparsity of publicly available genome data from much of the Caribbean limits a high-resolution phylogenetic reconstruction to determine outbreak origins, transmission pathways, and evolutionary histories, though sequencing efforts in the region are ongoing. Nevertheless, our analysis links the current outbreak in Cuba to the Brazil ECSA clade II (5) in accordance with recent analyses of travel-associated cases in Europe (7). Together with the placement of the uady-tam_2025, this suggests an expanding geographic range of the ECSA lineage into the Caribbean and potentially continental North America. In this scenario, strengthened regional collaboration for virus monitoring, entailing balanced genomic surveillance efforts across countries, will be necessary to better characterize CHIKV circulation across the Americas, including lineage distribution range, diffusion process, and timing of introduction events into different regions.

## Data Availability

Genome sequences are available in the NCBI GenBank database under accession numbers PX935460 and PX935461.
